# Metacognitive Therapy for People Experiencing Persistent Post-Concussion Symptoms Following Mild Traumatic Brain Injury: A Preliminary Multiple Case-Series Study

**DOI:** 10.1089/neur.2024.0076

**Published:** 2024-10-14

**Authors:** Johanne C. C. Rauwenhoff, Roger Hagen, Migle Karaliute, Odin Hjemdal, Leif Edward Ottesen Kennair, Stian Solem, Robert F. Asarnow, Cathrine Einarsen, Joar Øveraas Halvorsen, Stephanie Paoli, Simen Berg Saksvik, Hanne Smevik, Gøril Storvig, Adrian Wells, Toril Skandsen, Alexander Olsen

**Affiliations:** ^1^ Department of Psychology, Norwegian University of Science and Technology, Trondheim, Norway.; ^2^ NorHead—Norwegian Centre for Headache Research, Norwegian University of Science and Technology, Trondheim, Norway.; ^3^ Department of Psychology, University of Oslo, Oslo, Norway.; ^4^ Research Institute, Modum Bad, Vikersund, Norway.; ^5^ Department of Neurology and Clinical Neurophysiology, St. Olavs Hospital, Trondheim University Hospital, Trondheim, Norway.; ^6^ Division of Mental Health and Substance Abuse, Diakonhjemmet Hospital, Oslo, Norway.; ^7^ Department of Psychiatry and Biobehavioral Science, David Geffen School of Medicine, UCLA, Los Angeles, California, USA.; ^8^ Department of Psychology, UCLA School of Medicine, UCLA, Los Angeles, California, USA.; ^9^ Brain Research Institute, UCLA, Los Angeles, California, USA.; ^10^ Department of Neuromedicine and Movement Science, Norwegian University of Science and Technology, Trondheim, Norway.; ^11^ Clinic of Rehabilitation, St. Olavs Hospital, Trondheim University Hospital, Trondheim, Norway.; ^12^ Nidaros District Psychiatric Hospital, St. Olavs Hospital, Trondheim University Hospital, Trondheim, Norway.; ^13^ Department of Mental Health, Norwegian University of Science and Technology, Trondheim, Norway.; ^14^ Division of Psychology and Mental Health, School of Psychological Sciences, Faculty of Biology, Medicine and Health, The University of Manchester, Manchester, United Kingdom.; ^15^ Greater Manchester Mental Health NHS Foundation Trust, Manchester Academic Health Science Centre, Manchester, United Kingdom.

**Keywords:** metacognitions, metacognitive therapy, mild traumatic brain injury, post-concussion symptoms

## Abstract

After mild traumatic brain injury (mTBI), a subgroup of individuals experience persistent post-concussion symptoms (PPCS) that include headaches, cognitive difficulties, and fatigue. The aim of this preliminary study was to investigate possible effects associated with metacognitive therapy (MCT) on PPCS, maladaptive coping strategies, and positive and negative metacognitive beliefs following mTBI. A pre–post design supplemented with single-case A–B replication series to assess potential MCT mechanisms was used. Of the nine participants who received MCT, all experienced a decrease in PPCS, which constituted a reliable improvement for eight participants. For eight participants (we could calculate effect sizes for eight out of nine participants), moderate to very large decreases in maladaptive coping styles and positive and negative metacognitive beliefs were observed. However, based on visual analyses, participants 6, 8, and 9 show a downward baseline trend regarding MCT mechanisms that may have persisted into the intervention phase. No adverse events were reported. In conclusion, MCT was associated with improvements in PPCS and unhelpful psychological mechanisms, but caution is required in interpreting this association. Future research using formal single-case replication on symptom measures and randomized controlled trials appears to be justified.

## Introduction 

Following a mild traumatic brain injury (mTBI), people can experience an array of different post-concussion symptoms (PCS), including headaches, cognitive complaints, emotional problems, and fatigue.^1^ For most people, these complaints resolve within the first 3 months following the mTBI. However, a subgroup continues to experience PCS after 3 months,^2–4^ known as persistent PCS (PPCS). PPCS significantly impacts daily functioning, causes distress, and reduces quality of life.^5^ The etiology of PPCS remains unclear but might be understood from a biopsychosocial perspective,^6^ recognizing a complex interplay between factors related to preinjury vulnerabilities (e.g., pain, poor sleep quality, and working less than full time), acute physiological processes (e.g., inflammatory responses), and post-injury factors (e.g., cognitive functioning, resilience, coping strategies, psychosocial support or stressors, and psychological processes including maladaptive beliefs).^6–11^

There is a growing interest in researching psychological interventions for PPCS with cognitive training, psychoeducation, cognitive behavioral therapy, third-wave therapies, and graded return to activity showing possible positive effectiveness.^12,13^ However, the evidence is limited and of low quality, and more research is needed.^12,14,15^

Metacognitive Therapy (MCT) is a transdiagnostic treatment that has shown promising results in a range of different patient groups, including people experiencing major depression,^16,17^ generalized anxiety,^18,19^ obsessive-compulsive disorder,^20^ post-traumatic stress,^21^ and anxiety and depression symptoms in people with cardiovascular disease.^22^ So far, MCT has not been studied in people with PPCS. MCT aims to change the way people think about their thinking and how they respond to their thoughts by targeting metacognitive beliefs and mental regulation strategies.^23^ MCT is based on the Self-Regulatory Executive Function (S-REF) model,^24^ which posits that psychological disorders are maintained by perseverative thinking and attentional processes—the Cognitive Attentional Syndrome (CAS). CAS consists of worry, rumination, and threat monitoring, as well as maladaptive coping strategies such as avoidance, thought suppression, and sedation.^23^ CAS is regulated by a metacognitive system that is comprised, in part, of metacognitive beliefs, which are the beliefs people have about their thinking. MCT aims to reduce CAS and modify erroneous metacognitive beliefs to enable the development of flexible reactions to negative thoughts, feelings, and sensations.^25,26^ Techniques employed in MCT include the attention training technique, detached mindfulness, and personal experiments of postponing rumination and worry.^26^ These strategies are embedded within a meta-level therapist–patient discourse that is configured to modify different components within the metacognitive control system.^26^

PPCS-specific metacognitive beliefs could be positive, such as “If I think a lot about my symptoms, I will understand them and be able to cope with them,” or negative, such as beliefs about uncontrollability (e.g., “I cannot stop worrying about my symptoms”) and harm (e.g., “Worrying will make me sick”). These metacognitive beliefs can influence CAS processes^23^ and might explain why some people with PPCS have difficulty adapting and start catastrophize about their symptoms.^27–29^ Maladaptive coping strategies can include fear avoidance and endurance behavior, which are both related to PPCS.^30,31^ Hypervigilance toward physical symptoms is present in people with somatic distress,^32,33^ which in the MCT model is viewed as a threat-monitoring coping strategy, and people with PPCS are likely to closely monitor and scan their bodies and cognitive functioning for any new or worsening symptoms.

Given that an important treatment target is to alter metacognitive beliefs and reduce CAS activity, MCT might therefore be particularly suited for people with PPCS. Furthermore, it is a transdiagnostic approach, and there are indications that metacognitive beliefs play a role in maintaining both psychological and somatic symptoms.^34,35^ A review by Keen et al.^34^ found that maladaptive metacognitive beliefs are related to more somatic distress and physical symptoms (such as pain and fatigue) in people with health anxiety, cyberchondria, chronic pain, fibromyalgia, chronic fatigue syndrome, irritable bowel syndrome, and somatic symptoms. While PPCS was not included in the review of Keen et al.,^34^ the conditions that were discussed show similarities with PPCS as there is a significant overlap between PPCS and symptoms of, for instance, chronic pain.^36^ The possible mechanisms underlying the development and maintenance of these symptoms are likely similar and can be best understood from a biopsychosocial perspective.^37,38^ Furthermore, metacognitions are positively related to anxiety, depression, and quality of life in people with chronic health conditions such as multiple sclerosis, chronic pain, stroke, and chronic fatigue syndrome.^39^ In addition, positive metacognitive beliefs about worrying predict, together with headache frequency, the chronicity of migraine,^40^ which is a frequently occurring symptom following mTBI. Last, MCT may positively influence both biological and social mechanisms. It can enhance adaptive behaviors, improving activity balance and participation,^17^ which in turn can reduce PPCS symptoms and enhance overall functioning.

The primary aim of this preliminary study was to investigate the effects associated with MCT on PPCS following mTBI. Furthermore, we explored whether maladaptive coping strategies and positive and negative metacognitive beliefs (CAS processes) changed during and after treatment. Finally, we investigated the feasibility and acceptability of the study design, considering future large-scale randomized controlled trials.

## Methods

### Participants

Patients were recruited from a larger cohort study; the Trondheim mTBI follow-up study,^41^ and from an outpatient brain injury rehabilitation clinic at St. Olavs Hospital, Trondheim University Hospital. The inclusion criteria for the cohort were: age between 16 and 60 years, having sustained an mTBI, and having PCS (according to the International Statistical Classification of Diseases and Related Health Problems 10th Revision and as evaluated by a medical doctor) for more than 6 months after the mTBI. The World Health Organization criteria for mTBI were used: Glasgow Coma Scale score 13–15 at presentation, loss of consciousness (LOC) <30 min, and/or post-traumatic amnesia (PTA) <24 h.^42^

The exclusion criteria for the study were: nonresidency in Norway or non-fluency in Norwegian; major other trauma with a high risk of disability lasting more than 3 months; major incidental intracranial findings in acute magnetic resonance imaging (MRI) (e.g., cyst, tumor, malformation, infarctions); severe psychiatric, neurological or medical disease (e.g., psychotic disorders, bipolar disorder, ongoing severe depressive episode); personality disorders affecting adherence to the research protocol; alcohol/drug abuse affecting adherence to the research protocol; intellectual disability, autism or other severe developmental disorders; prior complicated-mild, moderate, or severe TBI; stroke or other acquired brain injuries; progressive neurological disorders (e.g., Parkinson’s disease, MS); and advanced cancer, heart or respiratory disease or other somatic diseases that interfere with function. In addition to the exclusion criteria already employed in the cohort study, patients with co-occurring psychiatric disorders that necessitated referral to specific treatment according to existing guidelines were excluded from the current intervention study.

Ethics approval for the study was granted by the Regional Committee for Medical Research Ethics Central Norway (2015/1456) and performed in accordance with the Helsinki Declaration. The study was registered in ClinicalTrials.gov (NCT02690584) on February 24, 2016 https://classic.clinicaltrials.gov/ct2/show/NCT02690584.

### Outcome measures

#### Primary outcome measure

The Rivermead Post-concussion symptom Questionnaire (RPQ) total score was used to assess the severity of somatic, cognitive, and emotional PCS.^43^ The scale consists of 16 items that are rated on a five-point Likert scale, with 0 representing no experience at all and 4 representing the symptom being a severe problem. The total score ranges from 0 to 64. The Norwegian version of the RPQ has been found to be comparable to the English version.^44^

#### Process measure

The Cognitive Attentional Syndrome scale (CAS-1) was used to measure the levels of CAS.^26^ The CAS-1 consists of 16 items. Three subscales were calculated. The first eight items, measured on a 0–8 scale, assess strategies with which people cope with negative feelings or thoughts. The next four items measure positive metacognitive beliefs on a 0–100 scale, and the last four items measure negative metacognitive beliefs, also on a 0–100 scale. The CAS-1 has satisfactory reliability and validity.^45^

### Design and procedure

The study had a baseline-controlled repeated measures design in which all participants were allocated to the same intervention and assigned to a fixed 2-week baseline period before the commencement of the intervention. The CAS-1 was completed during the baseline period at three time points: 2 and 1 week before the start of the intervention, and just before the first treatment session. Furthermore, participants completed the CAS-1 before the start of each therapy session (i.e., the treatment phase), and there were assessments at pre-treatment, post-treatment, 10-week follow-up, and 6-month follow-up where participants completed both the RPQ and CAS-1. The RPQ was not completed at the baseline measurements and during the treatment phase, as this was deemed too burdensome for the participants.

There were some deviations from the original study protocol. First, fewer participants were recruited. We initially aimed to recruit a sample size of 20 patients. This was due to challenges related to patient recruitment and study logistics, including periods of mismatch in the availability of patients, therapists, and study personnel, as well as patient drop-out at the end of the inclusion period due to the COVID-19 pandemic.

Second, given the smaller sample size and missing data, the original analysis plan was altered. Instead of analyzing the data on a group level, it was decided to analyze the data on an individual level, using single-case repeated measurements of process variables. Single-case studies have been extensively used to evaluate neuropsychological treatments.^46^ Single-case research is also included in evidence standards, for instance, the American Psychological Association,^47,48^ and due to the rapidly developing statistical techniques, such as meta-analytic procedures, results can be generalized to specific populations.^49^ Given the small number of participants and use of baseline measurements, visual analysis of time-series data on an individual level can yield more information and provide additional support for treatment-related effects than analyzing the data on a group level as originally planned.

### Intervention

The intervention consisted of 10 one-on-one MCT sessions over 10 weeks, each lasting 45–60 min. The treatment was based on and used the methods described in the MCT manual by Wells.^26^ This transdiagnostic protocol deemphasizes diagnostic labels and focuses instead on modifying specific mechanisms, namely, positive and negative metacognitions and the use of worry, rumination, self-focused attention, and unhelpful coping behaviors to regulate emotions. Following each session, the participants received homework exercises to practice MCT techniques at home. Therapy sessions were provided at the outpatient clinic of the Department of Psychology at the Norwegian University of Science and Technology, by four experienced MCT-Institute registered (Levels I and II) therapists.

### Analyses

The reliable change index (RCI) was used to determine whether a change in the primary outcome (RPQ) was a reliable change between the pre- and post-treatment and follow-up assessments. This was done using the formula of Jacobson and Truax^50^ using the standard deviation and reliability coefficients from Medvedev et al.^51^ A reliable change is found when the outcomes are *z* < −1.96 or *z* >1.96. Pre-treatment scores of the RPQ were compared with post-treatment and follow-up scores. Furthermore, we assessed whether participants scored under the cutoff score on the RPQ at the last follow-up assessment; a cutoff score of 12 was used.^52^ Furthermore, as supplementary (exploratory) analyses, the RCI was calculated for each PCS separately.

For each participant, levels of CAS activity (coping strategies, negative and positive metacognitive beliefs) were plotted graphically to support visual analyses. Visual analyses were conducted following the recommendations of Ledford and Gast.^53^ Horizontal lines are depicted to observe changes in the average score between phases (baseline phase and treatment + follow-up phase). The trend was determined by the slope and direction of the best-fitting straight line for each phase using the Split Middle Method. Trend stability was defined by a stability window ±25% of the trend line. To acquire an effect size, Tau-U was calculated for the three subscales of the CAS-1. The baseline phase was compared to the treatment + follow-up measurements, as we expected that the effect of MCT would continue after the termination of therapy. Tau-U is derived from Kendall’s Rank Correlation and the Mann–Whitney *U* test and is an often-used effect size in single-case research.^54,55^ Tau-U has several advantages over other effect size measures of which the most important is that it performs reasonably well with autocorrelated data and has high sensitivity and power in short time series.^55^ The baseline was checked for trends, and Tau-U was adjusted for baseline trend where necessary. Tau-U analyses were also conducted, excluding the two follow-up assessments to determine if the two follow-up assessments influenced the results. Tau-U was interpreted as follows: 0.20 small effect, 0.20–0.60 moderate, 0.60–0.80 large, and above 0.80 a large to very large effect.^56^ Tau-U was calculated using the SingleCaseES package in R.^57^ Tau-U was not calculated if there was only one measurement in a phase.

## Results

### Recruitment and procedure

Participants were included between February 2016 and April 2019. In total, 15 people with PPCS after mTBI were included. For four participants, therapy was terminated after one or two sessions. Therapists indicated that these participants did not have a clear request for help, were not treatment seeking, and that levels of CAS activity (particularly worry and rumination) were too low to continue with treatment. One participant was lost to follow-up during treatment, and one participant’s data were lost. As a result, nine participants are included in the analyses of this study.

There was considerable between-subject variation in actual assessment points. The time between the first two baseline assessments varied from 7 to 33 days, and the time between the second and last baseline assessment varied from 5 to 21 days. The time between the pre- and post-treatment assessments varied from 11 to 24 weeks, between the post-treatment and the 10-week follow-up ranged between 9 and 17 weeks, and between the 10-week and 6-month follow-up between 12 and 23 weeks.

### Participants

Demographic characteristics can be found in Table 1. The average age of participants was 40.22 (*SD* = 10.24). Five of the nine participants were female and all but one were highly educated. Participants were on average 21.67 months post-injury (*SD* = 9.15). For three participants a traffic accident was the cause of the mTBI, for three a fall (one from their own height and two greater), for two a sports accident, and for one a collision. Glasgow Coma Scale score was 15 for eight participants and for one unknown. Three participants had no LOC, three 5 min or less, one between 5 and 15 min, and for two this was unknown. Six participants had post-traumatic amnesia of <1 h, one between 2 and 3 h, and two experienced no PTA. All participants had normal MRI and computerized tomography scans, except for one participant where microhemorrhages were found on the MRI scan.

**Table 1.** Demographic Information

	PP1	PP2	PP3	PP4	PP5	PP6	PP7	PP8	PP9
Age	56	35	35	25	48	52	40	41	30
Gender	Female	Male	Female	Male	Female	Male	Male	Female	Female
Marital status	Living alone	Living together	Married	Living together	Married	Married	Living alone	Married	Living together
Years of education	18	18	18	17	18	12	16	17	19
Work status	80% sick leave	50% sick leave	Works 100%	Works 100%	Works 100%	Works 40%	20% sick leave	Works 60% and studies	100% sick leave

### Primary outcome measure

The RPQ scores at baseline ranged between 13 and 50. All participants showed lower RPQ scores at the follow-up measures compared to baseline as can be seen in Table 2. Participants reported improvements ranging from 10 to 19 points on the RPQ, with an average improvement of 13.33 points. All but one participant met the criteria for reliable improvement at the last follow-up assessment (an improvement of 9 points on the RPQ was a reliable improvement). At their last follow-up assessment, four participants scored below the cutoff of 12 on the RPQ. Supplementary analyses investigating PCS symptoms separately indicated that on all symptoms, except for double vision, at least one participant showed reliable improvements. The reliable change per symptom can be found in Supplementary Table S1.

**Table 2.** Reliable Change per Participant on the Rivermead Post-Concussion Questionnaire Scores

	PP1 (rCI)	PP2 (rCI)	PP3 (rCI)	PP4 (rCI)	PP5 (rCI)	PP6 (rCI)	PP7 (rCI)	PP8 (rCI)	PP9 (rCI)
Pre-treatment	34	26	50	16	24	29	29	13	19
Post-treatment	22 (2.74^a^)	14 (2.76^a^)	24 (5.99^a^)	16 (0)	17 (1.61)	24 (1.15)	17 (2.76^a^)	0 (2.99^a^)	13 (1.38)
Follow-up 10 weeks	—	14 (2.76^a^)	—	4 (2.76^a^)	11 (2.99^a^)	25 (0.92)	20 (2.07^a^)	2 (2.53^a^)	16 (0.69)
Follow-up 6 months	19 (3.46^a^)	7 (4.38^a^)	—	6 (2.30^a^)	10 (3.23^a^)	17 (2.76^a^)	19 (2.30^a^)	—	—

The table includes raw scores with the reliable change index, that is, *z*-scores, between brackets.

^a^ Reliable decrease between this measuring point and the pre-treatment measuring point.

rCI, reliable change index.

### Process measure (CAS-1)

Table 3 shows the phase characteristics and Tau-U scores for each participant for the three CAS-1 subscales. Based on visual analyses, participants 6, 8, and 9 show a downward baseline trend that may have persisted into the intervention phase.

**Table 3.** Phase Characteristics and Tau-U of the Repeated CAS-1 Scores

Participant	No. of baseline measurements	Mean (SD) baseline	Trend (trend stability %) baseline	No. of treatment + follow-up measurements	Mean (SD) treatment + follow-up	Trend (trend stability %) treatment + follow-up phase	Tau-U
CAS coping							
PP1	2	10.5 (0.71)	+ (100)	11	4.73 (3.10)	− (27)	0.91
PP2	3	15(4)	− (67)	12	7.67 (6.77)	− (25)	0.58
PP3	1	—	—	10	14 (11.26)	− (90)	—
PP4	3	2.67 (2.52)	− (67)	13	0.46 (0.78)	0 (85)	0.54
PP5	3	14.67 (4.04)	− (67)	7	4.57 (1.9)	0 (57)	0.95
PP6	3	13.33 (5.77)	− (67)	3	0 (0)	0 (100)	0.78
PP7	3	18.33 (4.16)	− (67)	12	15.17 (4)	− (75)	0.47
PP8	3	10.67 (4.16)	− (67)	10	1.5 (1.65)	− (20)	0.97
PP9	3	16.33 (8.02)	− (100)	11	2.82 (1.78)	− (55)	0.91
CAS positive metacognitions							
PP1	2	90 (0)	0 (100)	12	46.67 (31.93)	− (67)	0.75
PP2	3	93.33 (23.09)	− (100)	12	26.67 (35.51)	− (0)	0.83
PP3	1	—	—	10	52 (63.74)	− (10)	—
PP4	3	50 (30)	− (67)	13	59.23 (18.91)	0 (69)	−0.21
PP5	3	130 (36.06)	− (100)	8	25 (17.73)	− (63)	0.88
PP6	3	83.33 (23.09)	− (67)	3	0 (0)	0 (100)	0.78
PP7	3	130 (36.06)	+ (67)	12	93.33 (30.55)	− (92)	0.69
PP8	3	40 (51.96)	− (67)	10	9 (25.14)	0 (80)	0.73
PP9	3	163.33 (5.77)	+ (100)	11	33.64 (28.03)	− (36)	1.06
CAS negative metacognitions							
PP1	2	130 (0)	0 (100)	12	95.42 (38.11)	− (58)	0.67
PP2	3	133.33 (20.82)	− (100)	12	60 (60.90)	− (75)	0.56
PP3	1	—	—	10	130 (99.22)	− (80)	—
PP4	3	93.33 (25.17)	+ (67)	13	30.77 (27.53)	− (23)	0.95
PP5	3	193.33 (15.28)	− (100)	8	38.75 (6.41)	0 (100)	0.88
PP6	3	83.33 (75.06)	− (67)	3	0 (0)	0 (100)	0.78
PP7	3	216.67 (5.77)	+ (100)	12	131.67 (52.02)	− (83)	1.06
PP8	3	140 (40)	− (67)	10	20.50 (11.17)	+ (20)	0.97
PP9	3	103.33 (15.28)	− (67)	11	19.09 (20.71)	− (27)	0.97

CAS-1, Cognitive Attentional Syndrome scale; SD, standard deviation; 0, zero-accelerating trend line; +, accelerating trend line; −, decelerating trend line.

**FIG. 1.**
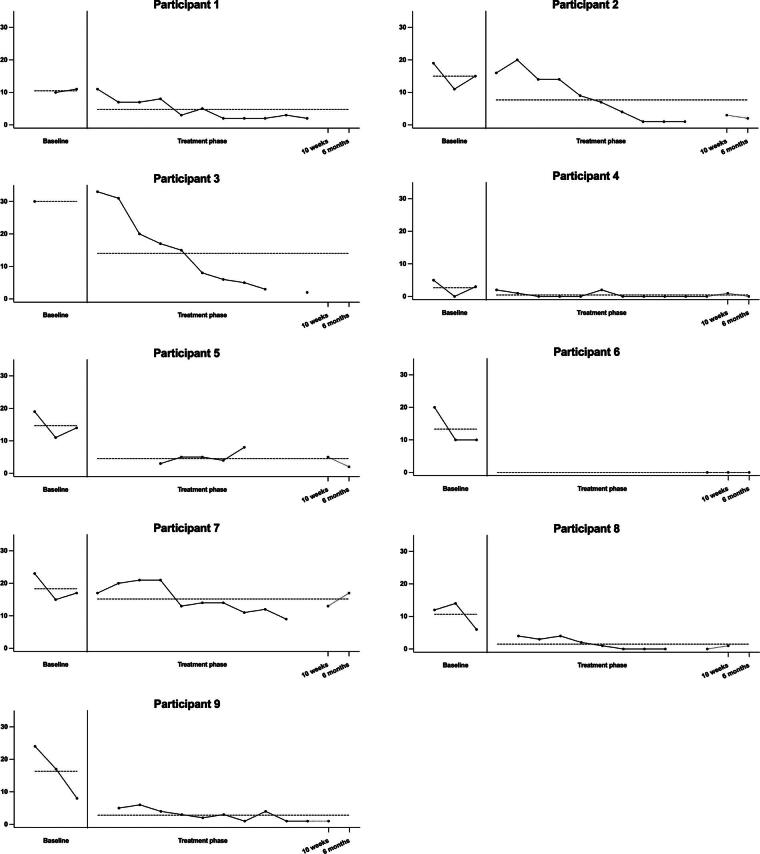
Levels of maladaptive coping per participant. The dotted vertical line represents the start of the intervention, and the dotted horizontal line represents the average score on the baseline and treatment + follow-up phases.

**FIG. 2.**
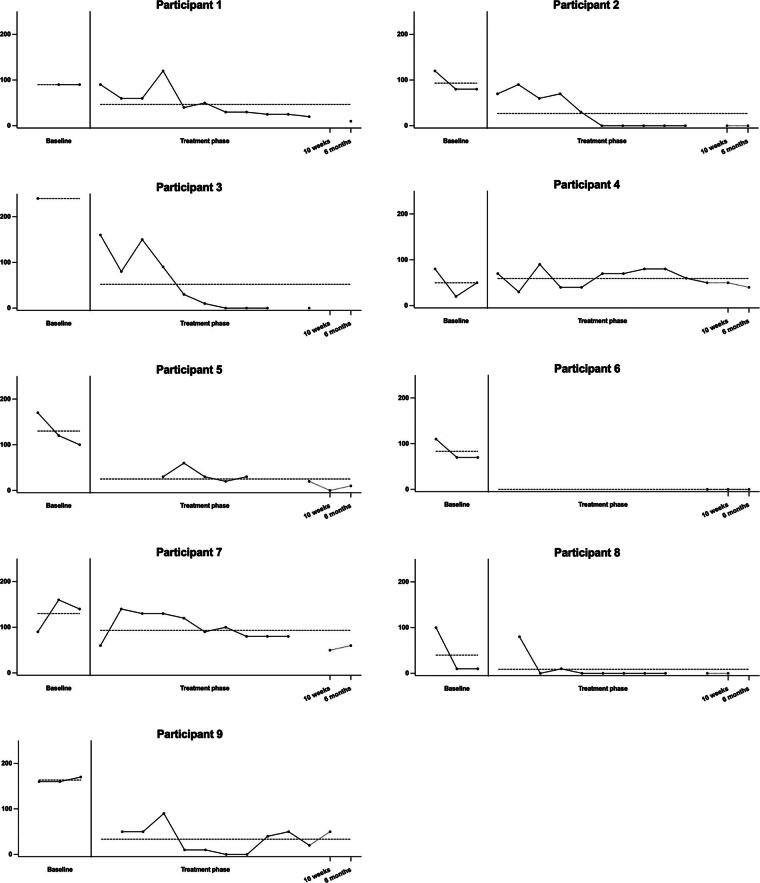
Levels of maladaptive positive metacognitions per participant. The dotted vertical line represents the start of the intervention, and the dotted horizontal line represents the average score on the baseline and treatment + follow-up phases.

**FIG. 3.**
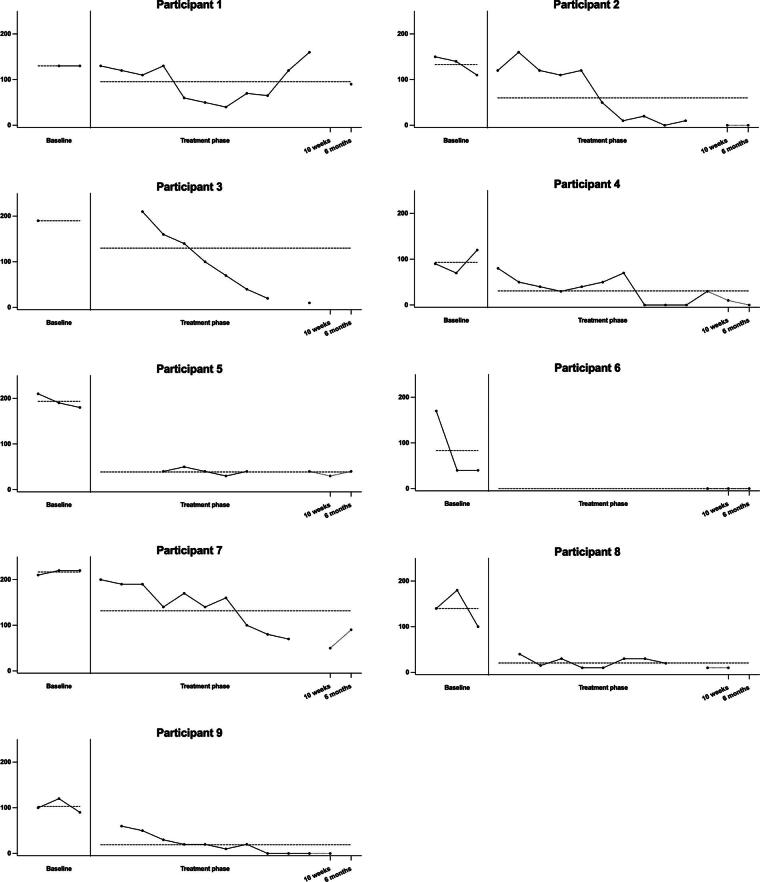
Levels of maladaptive negative metacognitions per participant. The dotted vertical line represents the start of the intervention, and the dotted horizontal line represents the average score on the baseline and treatment + follow-up phases.

None of the baseline trends were significant; therefore, Tau-U was not corrected for the baseline trend. On the CAS-1 coping scale (Fig. 1), all participants had lower scores in the treatment + follow-up phase compared to the baseline phase. Four participants showed a very large decrease in CAS-1 scores, for three there was a moderate decrease, and for one participant a large decrease as calculated with Tau-U. On the CAS-1 positive metacognitions (Fig. 2), all but one participant had lower scores in the treatment + follow-up phase compared to the baseline phase. Four participants showed a large decrease in CAS-1 scores, and for three participants this was a very large decrease. One participant showed a large increase. On the CAS-1 negative metacognitions (Fig. 3), all participants showed a decrease in scores between the baseline and treatment + follow-up phase, for five participants this was a very large decrease, for two participants this was a large decrease, and for one a moderate decrease. Tau-U analyses were also conducted, excluding the two follow-up assessments for participants who still had enough data points to determine if the two follow-up assessments influenced the results. The analyses showed no differences in the results.

## Discussion

In the current preliminary study, the effects associated with MCT for people who experience PPCS following mTBI were examined. We found that all nine participants experienced a decrease in PPCS, which was a reliable improvement for eight participants. At the 6-month follow-up, participants reported improvements ranging from 10 to 19 points on the RPQ, with an average improvement of 13.33 points. Previous RCTs of cognitive behavioral therapy for people with PPCS have found an average improvement of 1.4 points^58^ (*n* = 90), 7.8^52^ (*n* = 46), and 15.6^59^ (*n* = 28) points on the RPQ. The study with the largest effect^59^ was conducted in the early stage following mTBI (<3 months post-injury), when higher rates of spontaneous recovery were expected, and treatment was utilized as a preventative intervention. The reduction of PPCS in our study therefore favorably compares to previous studies investigating psychological interventions in this group. However, many participants who experienced a reduction in symptoms still experienced considerable symptom burden (4 participants with an RPQ <12). As anticipated, there was considerable heterogeneity among participants regarding the types of symptoms reported at baseline. Supplementary analyses revealed that reliable improvements, as determined by the RCI, were observed for most individual symptoms, including somatic symptoms such as headache and noise sensitivity.

Eight participants (we could calculate effect sizes for 8 out of 9 participants) experienced a moderate to very large decrease in maladaptive coping styles and positive and negative metacognitive beliefs, except for participant 4 who experienced an increase in positive metacognitions, although their initial CAS-1 scores were not very high.^60^ Furthermore, participants 6, 8, and 9 had decreasing baseline trends that may have persisted into the intervention phase.

While these changes in PCS symptoms and the mechanism measures are consistent with the S-REF model,^26^ it is important to note that we cannot conclude that the changes were caused by the intervention or that the primary treatment effect was mediated by the proposed process variables. However, a correspondence between symptom and process measures can be observed in six out of eight participants. These participants experienced both reliable improvements in PCS and moderate to very large improvements in CAS activity. However, there are some noteworthy discrepancies. In the case of one participant, reliable improvements in PCS were not observed, but substantial improvements in unhelpful coping styles and metacognitive beliefs were reported.

For four out of the 16 recruited patients, therapy was discontinued because therapists indicated that these participants did not have a clear request for help, were not treatment seeking, and that levels of CAS activity (particularly worry and rumination) were low and further treatment was not indicated. Levels of worry, rumination, and PPCS-specific metacognitions might be considered in future treatment selection. In addition, the relationship between CAS and PPCS and other needs expressed by the patient can help to identify patients who might benefit most from MCT. By exploring personalized approaches, we can better understand how to match therapeutic interventions to the characteristics, needs, and circumstances of each person, ultimately optimizing treatment outcomes and improving the overall effectiveness of therapy.^61^

The results of the present study are encouraging and support future studies of MCT in PPCS. Such studies should aim to examine the effect of treatment in randomized controlled trials and examine a wider range of symptoms, including adjustment, cognitive functioning, and quality of life. A strength of the present study is that we tried to maintain treatment fidelity and theoretical coherence by following a specific treatment manual. However, the current treatment protocol did not include all MCT methods. A technique that might be suitable for people who adopt an avoidant coping style toward situations that could worsen PPCS is metacognitively delivered exposure. This approach involves modifying the individual’s attentional and thinking patterns in conjunction with exposure to the feared stimuli or situations.^25^ People can learn that in fearful situations they are still able to control worry, and PCS might not increase.

Furthermore, the treatment did not include a range of idiosyncratic techniques, which enhances treatment fidelity by reducing therapy drift and the risk of using incompatible methods. However, it may not fully meet the psychological needs of all patients. Future studies should examine these psychological needs and aim to determine the broader effects of MCT or areas in which MCT might be used to supplement standard practice such as the provision of psychoeducation. For people with chronic pain, including a session on psychoeducation is regarded as a “gold standard” in psychological treatment.^62^ Psychoeducation can have a positive effect,^14^ as it can normalize PCS following an mTBI, enhance the patients’ understanding of the complicated nature of mTBI, and help people understand why a psychological intervention might be useful for physical symptoms.^63^ However, the metacognitive model emphasizes caution in the judicious use of psychoeducation because information can be processed by patients in a manner not consistent with developing internal long-term self-regulation of worry (e.g., a dependence on external information to regulate repetitive negative thinking can be strengthened). Lastly, considering the multifaceted nature of PPCS, it is logical to approach treatment from a biopsychosocial perspective.^10^ If the efficacy of MCT and its principles is confirmed, we could examine how MCT can become part of a comprehensive and multidisciplinary treatment approach for individuals experiencing PPCS, encompassing interventions targeting social and biological factors.

There were several challenges to patient recruitment and adherence that should be considered when planning future trials. Initially, our approach involved recruiting patients who experienced PPCS for over a year from an ongoing observational cohort study. However, contrary to our expectations, a significant number of patients reporting a high symptom load one year after the injury did not express a need for treatment. Consequently, we had to modify the recruitment strategy to include patients from an outpatient rehabilitation clinic, and we adjusted the cutoff from 1 year to 6 months. mTBI is a heterogeneous condition, and in addition to considering injury-related inclusion/exclusion criteria, both recruitment to and, adherence in future trials may benefit from a more thorough assessment of whether patients are actively treatment seeking.

This study has several strengths. To our knowledge, this is the first study researching the effects associated with MCT in people who experience PPCS following mTBI. In addition, the study included both outcomes related to symptomatology as well as outcomes related to underlying treatment mechanisms. Last, no adverse events occurred for any of the participants.

Given the lack of a control group, we cannot control for changes observed that are due to regression to the mean, recovery over time, or placebo effects. Furthermore, participants for whom the treatment was prematurely discontinued were lost to follow-up, leading to missing data. The current sample consists only of participants who completed the intervention, and the results may therefore be subject to a sampling bias. The baseline for most participants consisted of three measurements, however, a minimum of five measurements per phase is recommended by some,^64^ but others state that a minimum of three data points suffice.^65^ However, since participant 1 has a baseline with 2 data points and participant 3 has a baseline with 1 data point, the results of these participants should be interpreted with caution. Future single-case studies should include longer and more stable baselines that also include repeated measurements of PPCS. Furthermore, there was a wide variability in the actual time between measurements. It is unclear whether and how this influenced the results. Overall, there is generally quite a lot of missing data in the study.

In conclusion, MCT was associated with PPCS and CAS improvements and may hold promise as a treatment option for people experiencing PPCS following mTBI. More research, in the form of adequately powered randomized controlled trials and formal replication with single-case studies, appears to be justified.

## Abbreviations Used

CAS: Cognitive Attentional Syndrome
CAS-1: Cognitive Attentional Syndrome scale
LOC: loss of consciousness
MCT: Metacognitive therapy
MRI: magnetic resonance imaging
mTBI: mild traumatic brain injury
PCS: post-concussion symptoms
PPCS: persistent post-concussion symptoms
PTA: post-traumatic amnesia
RCI: reliable change index
RPQ: Rivermead post-concussion symptom questionnaire
S-REF model: Self-Regulatory Executive Function model
