# Supplementary material for: Metacognitive Therapy for People Experiencing Persistent Post-Concussion Symptoms Following Mild Traumatic Brain Injury: A Preliminary Multiple Case-Series Study
Source: Neurotrauma Rep. 2024 Oct 14;5(1):890–902. doi: 10.1089/neur.2024.0076 (PMC11491585; doi:10.1089/neur.2024.0076)
Supplement: Supplementary Table S1 [file neur.2024.0076_supplementary_table_s1.pdf]

Supplementary materials

Tabel 1. Reliable changes per symptom on the Rivermead Post-concussion Questionnaire

	PP1	PP2	PP3	PP4	PP5	PP6	PP7	PP8	PP9
**Headache**									
Pre-treatment	4	1	4	3	2	3	2	0	2
Post-treatment	2 (2,31*)	0 (1,16)	3 (1,16)	2 (1,16)	1 (1,16)	3 (0,00)	1 (1,16)	0 (0,00)	3 (-1,16)
Follow-up 10 weeks	-	0 (1,16)	-	2 (1,16)	1 (1,16)	3 (0,00)	2 (0,00)	2 (-2,31*)	3 (-1,16)
Follow-up 6 months	2 (2,31*)	0 (1,16)	-	1 (2,31*)	0 (2,31*)	2 (1,16)	0 (2,31*)	-	-
**Dizziness**									
Pre-treatment	4	1	4	0	2	4	2	2	1
Post-treatment	2 (2,68*)	0 (1,34)	3 (1,34)	0 (0,00)	2 (0,00)	3 (1,34)	2 (0,00)	0 (2,68*)	0 (1,34)
Follow-up 10 weeks	-	0 (1,34)	-	0 (0,00)	1 (1,34)	3 (1,34)	0 (2,68*)	0 (2,68*)	1 (0,00)
Follow-up 6 months	2 (2,68*)	0 (1,34)	-	1 (-1,34)	2 (0,00)	2 (2,68*)	2 (0,00)	-	-
**Nausea**									
Pre-treatment	0	0	1	0	1	2	0	0	0
Post-treatment	0 (0,00)	0 (0,00)	0 (2,28*)	0 (0,00)	0 (2,28*)	2 (0,00)	0 (0,00)	0 (0,00)	0 (0,00)
Follow-up 10 weeks	-	0 (0,00)	-	0 (0,00)	0 (2,28*)	2 (0,00)	0 (0,00)	0 (0,00)	0 (0,00)
Follow-up 6 months	0 (0,00)	0 (0,00)	-	0 (0,00)	0 (2,28*)	2 (0,00)	0 (0,00)	-	-
**Noise sensitivity**									
Pre-treatment	2	2	4	0	3	2	0	0	3
Post-treatment	2 (0,00)	1 (2,43*)	3 (2,43*)	1 (-2,43*)	2 (2,43*)	1 (2,43*)	0 (0,00)	0 (0,00)	2 (2,43*)
Follow-up 10 weeks	-	1 (2,43*)	-	1 (-2,43*)	2 (2,43*)	2 (0,00)	0 (0,00)	0 (0,00)	2 (2,43*)
Follow-up 6 months	2 (0,00)	1 (2,43*)	-	0 (0,00)	2 (2,43*)	2 (0,00)	0 (0,00)	-	-
**Sleep disturbances**									
Pre-treatment	3	3	4	0	2	2	0	0	0
Post-treatment	1 (2,95)	2 (1,47)	4 (0,00)	1 (-1,47)	0 (2,95*)	1 (1,47)	1 (-1,47)	0 (0,00)	0 (0,00)
Follow-up 10 weeks	-	2 (1,47)	-	0 (0,00)	0 (2,95*)	1 (1,47)	1 (-1,47)	0 (0,00)	0 (0,00)
Follow-up 6 months	0 (0,00)	2 (1,47)	-	0 (0,00)	0 (2,95*)	0 (2,95*)	1 (-1,47)	-	-
**Fatigue**									
Pre-treatment	4	2	4	0	2	3	4	2	2
Post-treatment	3 (0,99)	2 (0,00)	3 (0,99)	1 (-0,99)	2 (0,00)	2 (0,99)	2 (1,99*)	0 (1,99*)	1 (0,99)
Follow-up 10 weeks	-	1 (0,99)	-	0 (0,00)	0 (1,99)	2 (0,99)	2 (1,99*)	0 (1,99*)	2 (0,00)
Follow-up 6 months	3 (0,99)	1 (0,99)	-	0 (0,00)	1 (0,99)	1 (1,99*)	2 (1,99*)	-	-
**Irritability**									
Pre-treatment	2	1	4	2	1	2	2	2	0
Post-treatment	1 (1,19)	1 (0,00)	1 (3,58*)	2 (0,00)	1 (0,00)	1 (1,19)	1 (1,19)	0 (2,39*)	0 (0,00)
Follow-up 10 weeks	-	1 (0,00)	-	0 (2,39*)	0 (1,19)	2 (0,00)	2 (0,00)	0 (2,39*)	0 (0,00)
Follow-up 6 months	0 (2,39*)	0 (1,19)	-	0 (2,39*)	1 (0,00)	0 (2,39*)	2 (0,00)	-	-
**Depression**									
Pre-treatment	0	1	2	1	1	2	3	1	0
Post-treatment	0 (0,00)	0 (1,67)	0 (3,33)	1 (0,00)	0 (1,67)	0 (3,33*)	1 (3,33*)	0 (1,67)	0 (0,00)
Follow-up 10 weeks	-	0 (1,67)	-	0 (1,67)	0 (1,67)	2 (0,00)	1 (3,33*)	0 (1,67)	0 (0,00)
Follow-up 6 months	0 (0,00)	0 (1,67)	-	0 (1,67)	0 (1,67)	0 (3,33*)	1 (3,33*)	-	-
**Frustration**									
Pre-treatment	2	1	3	1	2	3	4	2	0
Post-treatment	2 (0,00)	1 (0,00)	0 (5,15*)	1 (0,00)	2 (0,00)	2 (1,72)	2 (3,44*)	0 (3,44*)	0 (0,00)
Follow-up 10 weeks	-	1 (0,00)	-	0 (1,72)	1 (1,72)	2 (1,72)	2 (3,44*)	0 (3,44*)	0 (0,00)
Follow-up 6 months	0 (3,44*)	0 (1,72)	-	0 (1,72)	0 (3,44*)	2 (1,72)	3 (1,72)	-	-
**Forgetfulness**									
Pre-treatment	2	2	4	2	2	2	2	0	0
Post-treatment	2 (0,00)	1 (1,29)	2 (2,57)	1 (1,29)	1 (1,29)	2 (0,00)	2 (0,00)	0 (0,00)	0 (0,00)
Follow-up 10 weeks	-	2 (0,00)	-	0 (2,57*)	1 (1,29)	2 (0,00)	2 (0,00)	0 (0,00)	0 (0,00)
Follow-up 6 months	2 (0,00)	0 (2,57*)	-	0 (2,57*)	1 (1,29)	2 (0,00)	2 (0,00)	-	-
**Concentration**									
Pre-treatment	3	3	4	2	1	2	3	0	3
Post-treatment	2 (1,04)	1 (2,08*)	1 (3,13*)	1 (1,04)	1 (0,00)	2 (0,00)	2 (1,04)	0 (0,00)	2 (1,04)
Follow-up 10 weeks	-	2 (1,04)	-	0 (2,08*)	1 (0,00)	2 (0,00)	2 (1,04)	0 (0,00)	2 (1,04)

Follow-up 6 months	3 (0,00)	1 (2,08*)	-	1 (1,04)	0 (1,04)	2 (0,00)	2 (1,04)	-	-
**Taking longer to think**									
Pre-treatment	4	3	4	2	1	2	3	2	3
Post-treatment	3 (1,11)	2 (1,11)	2 (2,23*)	2 (0,00)	2 (-1,11)	2 (0,00)	2 (1,11)	0 (2,23*)	2 (1,11)
Follow-up 10 weeks	-	2 (1,11)	-	0 (2,23)	0 (1,11)	2 (0,00)	3 (0,00)	0 (2,23*)	2 (1,11)
Follow-up 6 months	3 (1,11)	0 (3,34*)	-	1 (1,11)	0 (1,11)	2 (0,00)	2 (1,11)	-	-
**Blurred vision**									
Pre-treatment	0	1	1	0	1	0	0	2	2
Post-treatment	0 (0,00)	1 (0,00)	1 (0,00)	1 (-1,37)	1 (0,00)	1 (-1,37)	0 (0,00)	0 (2,74*)	1 (1,37)
Follow-up 10 weeks	-	2 (-1,37)	-	0 (0,00)	0 (1,37)	2 (-2,74*)	3 (-4,11*)	0 (2,74*)	2 (0,00)
Follow-up 6 months	0 (0,00)	1 (0,00)	-	0 (0,00)	1 (0,00)	0 (0,00)	0 (0,00)	-	-
**Light sensitivity**									
Pre-treatment	3	2	3	3	2	0	2	2	3
Post-treatment	2 (1,29)	2 (0,00)	1 (2,57*)	2 (1,29)	1 (1,29)	1 (-1,29)	0 (2,57*)	0 (2,57*)	1 (2,57*)
Follow-up 10 weeks	-	2 (0,00)	-	1 (2,57*)	2 (0,00)	0 (0,00)	0 (2,57*)	0 (2,57*)	2 (1,29)
Follow-up 6 months	2 (1,29)	1 (1,29)	-	2 (1,29)	2 (0,00)	0 (0,00)	0 (2,57*)	-	-
**Double vision**									
Pre-treatment	0	1	1	0	0	0	0	0	0
Post-treatment	0 (0,00)	0 (1,88)	0 (1,88)	0 (0,00)	0 (0,00)	0 (0,00)	0 (0,00)	0 (0,00)	1 (-1,88)
Follow-up 10 weeks	-	0 (1,88)	-	0 (0,00)	0 (0,00)	0 (0,00)	0 (0,00)	0 (0,00)	1 (-1,88)
Follow-up 6 months	0 (0,00)	0 (1,88)	-	0 (0,00)	0 (0,00)	0 (0,00)	0 (0,00)	-	-
**Restlessness**									
Pre-treatment	1	2	3	0	1	0	2	0	0
Post-treatment	0 (1,09)	0 (2,17*)	0 (3,26*)	0 (0,00)	1 (0,00)	1 (-1,09)	1 (1,09)	0 (0,00)	0 (0,00)
Follow-up 10 weeks	-	0 (2,17*)	-	0 (0,00)	1 (0,00)	0 (0,00)	3 (-1,09)	0 (0,00)	0 (0,00)
Follow-up 6 months	0 (1,09)	0 (2,17*)	-	0 (0,00)	0 (1,09)	0 (0,00)	2 (0,00)	-	-

Note: Table includes raw scores with the reliable change index between brackets.
* Reliable change between this measuring point and the pre-treatment measuring point
